# The Power of *Drosophila melanogaster* for Modeling Neonicotinoid Effects on Pollinators and Identifying Novel Mechanisms

**DOI:** 10.3389/fphys.2021.659440

**Published:** 2021-04-21

**Authors:** Kiah Tasman, Sean A. Rands, James J. L. Hodge

**Affiliations:** ^1^School of Physiology, Pharmacology and Neuroscience, University of Bristol, Bristol, United Kingdom; ^2^School of Biological Sciences, University of Bristol, Bristol, United Kingdom

**Keywords:** neonicotinoids, honeybee, bumblebee, fruit fly, *Drosophila melanogaster*, insecticides, sub-lethal effects, behavior

## Abstract

Neonicotinoids are the most widely used insecticides in the world and are implicated in the widespread population declines of insects including pollinators. Neonicotinoids target nicotinic acetylcholine receptors which are expressed throughout the insect central nervous system, causing a wide range of sub-lethal effects on non-target insects. Here, we review the potential of the fruit fly *Drosophila melanogaster* to model the sub-lethal effects of neonicotinoids on pollinators, by utilizing its well-established assays that allow rapid identification and mechanistic characterization of these effects. We compare studies on the effects of neonicotinoids on lethality, reproduction, locomotion, immunity, learning, circadian rhythms and sleep in *D. melanogaster* and a range of pollinators. We also highlight how the genetic tools available in *D. melanogaster*, such as *GAL4/UAS* targeted transgene expression system combined with *RNAi* lines to any gene in the genome including the different nicotinic acetylcholine receptor subunit genes, are set to elucidate the mechanisms that underlie the sub-lethal effects of these common pesticides. We argue that studying pollinators and *D. melanogaster* in tandem allows rapid elucidation of mechanisms of action, which translate well from *D. melanogaster* to pollinators. We focus on the recent identification of novel and important sublethal effects of neonicotinoids on circadian rhythms and sleep. The comparison of effects between *D. melanogaster* and pollinators and the use of genetic tools to identify mechanisms make a powerful partnership for the future discovery and testing of more specific insecticides.

## Introduction

We are currently living through the sixth mass extinction event, the ‘Anthropocene extinction’ (Ceballos et al., 2017). Human activity and the associated changes in natural habitat availability and quality are causing mass reduction in biodiversity and abundance. Over the past 50 years insects, the most abundant and varied class of animal, have experienced significant reductions, documented by multiple longitudinal studies (Wagner, 2020). In Germany, there was a 76% decrease in flying insect biomass between 1989 and 2016 (Hallmann et al., 2017). A study in Puerto Rico found that between 1976 and 2012, 96% of ground insects had disappeared, in turn leading to reductions in other groups such as birds and frogs (Lister and Garcia, 2018). Globally it is estimated that 40% of insect species are at risk of extinction, with total insect biomass decreasing by 2.5% every year (Sánchez-Bayo and Wyckhuys, 2019). As seen in Puerto Rica, insects are a vital food source, and their loss endangers all species above them in the food chain. They also provide wider ecosystem services such as pest control, decomposition and pollination (Zhang et al., 2007). Insect pollination is required by 80% of wild plants, and 75% of crops and is worth over €153 bn to agricultural markets (Klein et al., 2007; Gallai et al., 2009; Potts et al., 2010). Most pollination is carried out by bees, perhaps the insect groups most adversely affected by the population losses due to neonicotinoids (Sánchez-Bayo and Wyckhuys, 2019). There are many additional factors which are contributing to these losses, largely the result of direct or indirect human activity, including climate change, habitat loss, diseases, parasites and invasive species (Goulson, 2019; Soroye et al., 2020). However, one of the major causes of pollinator loss is the intensive use of insecticides in agriculture (Gilburn et al., 2015; Wood and Goulson, 2017; Sánchez-Bayo and Wyckhuys, 2019; Wagner, 2020). Although insecticides are used with the aim of killing pest species, they are very often non-specific, affecting beneficial insects in the same way as pests. Globally, the most commonly used insecticides are the neonicotinoids, which have a good target species efficacy but exhibit a proven range of lethal and sub-lethal effects on beneficial insects such as bees. This makes the widespread use of neonicotinoids a major factor in pollinator decline (Gilburn et al., 2015; Wood and Goulson, 2017; Wagner, 2020).

## The Initially Hidden Problem of Neonicotinoid Sub-Lethal Effects

Many of the sub-lethal effects of neonicotinoids on beneficial insects were not identified until after their introduction and widespread use in the field from 1991. The current European Union (EU) screening process for pesticides looks at the effects of pesticides on the survival and development of the bumblebee colony during both acute and chronic exposure (European Food Safety Authority, 2013). During this exposure, any agrochemical causing a reduction in colony size (taken as a proxy for colony development) of more than 7% or a daily mortality rate within the colony exceeding 1.5 × that of controls are deemed unsafe. However chronic exposure tests only have to be carried out for a period of ten days, severely limiting the ability of these tests to identify the ongoing impact of sublethal effects on the colony. The EU guidance acknowledges this:

“Sublethal effects observed in individual bees have the potential to affect the development and the survival of the colonies. However, it is not possible with the information available to the working group to make a quantitative link between sublethal effects observed in first tier [initial] laboratory studies and effects on colonies. This could underestimate the risk.” (European Food Safety Authority, 2013).

Sublethal effects can indeed result in significant reductions in a colony’s capacity to reproduce, making them equally important to identify before a pesticide becomes widely available. For example, one study observed the development and reproduction of the colonies of the bumblebee *Bombus terrestris* over an 8 weeks period (Whitehorn et al., 2012). For the first 2 weeks, the colonies were exposed to a field-relevant dose of the most common neonicotinoid, imidacloprid and showed little difference from the control colonies in growth and development. However, for the following 6 weeks, with no further exposure to imidacloprid, these colonies grew significantly less than control colonies, resulting in their final mass being between 8 and 12% less than control colonies (Whitehorn et al., 2012). This reduction in growth, which is concerningly close to the level accepted as safe by the EU guidance, caused a decrease in queen production of 85–90%. The decreased growth of the colony was not from lethality but more likely from sub-lethal effects such as reduced foraging efficiency (Whitehorn et al., 2012; Tison et al., 2016), illustrating the importance of testing pesticides for sub-lethal effects prior to authorization.

## The Potential Use of High Throughput Assays in *Drosophila* to Pre-Screen for Sub-Lethal Effects

Screening pesticides for sublethal effects in pollinators can be time consuming. Toxicity screening in the medical and other industries is routinely carried out in simpler organisms to allow rapid, ethical and economical high throughput identification of adverse effects (Parasuraman, 2011). Perhaps a similar approach utilizing the model insect *D. melanogaster* could provide an initial, rapid and high-throughput *in vivo* behavioral platform to detect sublethal effects of any new insecticides. This could help determine its suitability for market prior to release into the environment and exposure to pollinators. *D. melanogaster* is a well-established model organism which has been used for modeling disease, drugs and neuroanatomy for decades (Pandey and Nichols, 2011; Ly et al., 2018; Higham et al., 2019). Extending this approach to other insects will be more insightful as *D. melanogaster*, the only model organism that is an insect, and should have similar molecular biology, metabolism and behaviors to other insect pollinators through shared ancestry. Furthermore the fly has the important advantage of being highly genetically tractable in addition to being small and having a short generation time and lifespan. This allows high throughput screening for lethal and sub-lethal effects of neonicotinoids which translate well to the effects seen in beneficial insects, making them ideal for drug development and for testing the suitability of a new insecticide for market (Jones et al., 2007; Kalajdzic et al., 2012; Charpentier et al., 2014; Denecke et al., 2017; Somers et al., 2017; Harrop et al., 2018; Fournier-Level et al., 2019; Homem et al., 2020; Martelli et al., 2020; Tasman et al., 2020, 2021).

*Drosophila melanogaster* has a relatively simple non-duplicated genome which is highly annotated and corresponds well to characterized neuroanatomy. For example its brain contains ∼135,000 neurons whose connections are understood at the level of serial electron microscopy allowing a publicly accessible whole connectome of the adult fly brain (Scheffer et al., 2020). Additionally, *D. melanogaster* is small, with a body mass around 100 × less than that of the honeybee *Apis mellifera* (Karan et al., 1998; Zoltowska et al., 2011), which is often used by the EU for testing pesticide safety, therefore many flies can be tested, or mutants generated and stored in a small lab. It also has a shorter developmental period (10 days compared to 21 days for a bee) and can lay up to 100 eggs a day, making it cheap to keep and assay at high quantities, and screen for rare mutations or effects. *D. melanogaster* has been used as the insect model organism for over 100 years and thus has high genetic tractability and a large number of well-established, reliable assays and reagents (e.g., gene mutations, antibodies etc.) for testing behavior and potentially any phenotype affected by insecticides.

The complete sequence of the fly genome was released in 2000 (Adams et al., 2000), and the genome remains exceptionally well annotated and curated by http://flybase.org/. There are many tools for influencing gene expression with mutations available for each of its 14,000 genes. These include null (knockout) alleles, over expression or knockdown (*RNAi*) transgenes of its endogenous genes also available through various stock centers and the labs who originally generated and published the reagents. Two tools used with great frequency are RNA interference (*RNAi*) lines and the *GAL4-UAS* system (Duffy, 2002; Heigwer et al., 2018). These can be used in conjunction to allow the silencing of genes in a spatio-temporal pattern, allowing detailed mapping of any genetic phenotype. Furthermore, the availability of a complete connectome of the whole fly brain and registered promoter lines allows mapping of phenotypes to single neurons (Eichler et al., 2017; Senturk and Bellen, 2018; Xie et al., 2018; Scheffer et al., 2020). Other commonly used genetic tools include loss of function mutations, over-expression of genes, expression of transgenes, and CRISPR-Cas9 technology for genome editing (Senturk and Bellen, 2018), for instance of neonicotinoid resistant mutations into nAChR genes in the fly genome (Zimmer et al., 2016; Homem et al., 2020). These provide tools for more in-depth exploration of the role of receptors targeted by neonicotinoids or other pesticides in the body and their mechanism of action. This can help highlight other potential sub-lethal effects to investigate and aid efforts for finding targets for more species-specific pesticides.

These genetic tools have already been widely put to use to investigate insecticide resistance in *D. melanogaster*, identifying genes that confer resistance to pesticides including neonicotinoids, spinosad, sulfoxaflor, pyrethroids, organophosphates, and DDT (Soderlund and Knipple, 2003; Ffrench-Constant et al., 2004; Watson et al., 2010; Bass, 2016; Zimmer et al., 2016; Perry and Batterham, 2018). While these examples illustrate the use of *D. melanogaster* as a model pest, it has so far been underutilized as a resource for the exploration of pesticide effects and modes of action in pollinators. Here we review the suitability and potential of *D. melanogaster* as an insect model organism for understanding the effects of neonicotinoids and future pesticides on beneficial insects.

## The Introduction of the “New Nicotine-Like” Insecticides Called Neonicotinoids

Neonicotinoids were originally branded as a new class of nicotinic acetylcholine receptor (nAChRs) agonist structurally similar to nicotine, hence their name. Nicotine has been used as a pesticide *via* tobacco extracts since at least 1690 (Tomizawa and Casida, 2004). The first commercial neonicotinoid, imidacloprid, was patented by Bayer in 1985 and brought to market in 1991 (Tomizawa and Casida, 2004; Nauen et al., 2008). Since then their popularity has grown steadily, overtaking previously wide-spread pesticides such as organophosphates. They are currently the most commonly used insecticides in the world, making up 24% of the total agrochemical market and 80% of the seed treatment market (Jeschke et al., 2011). Since the launch of imidacloprid, many other popular neonicotinoids have come onto the market, including clothianidin, thiamethoxam, thiacloprid and acetamiprid (Jeschke et al., 2011).

Neonicotinoids function as insecticides *via* their action as agonists at nAChRs (Tan et al., 2007). These receptors are found throughout the insect central nervous system, and their endogenous ligand, acetylcholine (ACh) is the main excitatory neurotransmitter there. Therefore, nAChRs are the predominant fast-synaptic neurotransmission system of the insect brain (Gauthier, 2010). When neonicotinoids bind to nAChRs, they can cause initial hyperexcitation of the neuron, however with prolonged presence of these agonists, especially at higher doses, they are thought to cause depolarizing block. This depolarizing block occurs *via* a number of steps starting with the neonicotinoid binding to the nAChR ligand gated ion channel and causing opening of its Na^+^ selective pore, resulting in depolarization of the neuron. Long-term depolarization causes voltage sensitive Na^+^ channels to become voltage inactivated, blocking the ability of neurons to fire action potentials. Next, the prolonged presence of the agonist causes nAChR desensitization, causing further neuronal inactivation (Tan et al., 2007). Clothianidin acts as a super-agonist of nAChRs, whilst imidacloprid and thiacloprid are both partial agonists. This likely explains the greater lethality to bees caused by clothianidin and thiamethoxam, which is a pro-drug for clothianidin (i.e., thiamethoxam gets rapidly metabolized into clothianidin) compared to imidacloprid (Nauen et al., 2003; Blacquiere et al., 2012). Similar sensitivities to the different neonicotinoids are seen in *Drosophila* with the non-banned thiacloprid being less toxic (Tasman et al., 2020, 2021). Furthermore the insecticides seem to act through a similar neuronal mechanism of action, causing initial hyperexcitability of neurons when applied to fly clock neurons in the whole brain (Tackenberg et al., 2020).

Neonicotinoids are used as a traditional spray insecticide, but they also make up the majority of the seed treatment market (Jeschke et al., 2011). Due to their high solubility and their systemic nature, neonicotinoids are absorbed by treated plants and appear in every tissue (Bonmatin et al., 2015). While this increases the efficacy of the insecticide against sap-sucking pests (Castle et al., 2005), it also means that the pollen and nectar of the plants contain significant concentrations of neonicotinoid. Analysis of crops has shown that nectar and pollen contain 1–51 μg/L for seed treated crops and 61–127 μg/L for sprayed crops (Goulson, 2013). These concentrations are sufficient to cause sub-lethal effects; doses as low as 1 μg/L have been shown to have behavioral effects in non-target species (Laycock et al., 2012). Insects can be exposed to higher doses shortly after spraying or when treated seeds are being sown, due to the high neonicotinoid concentration in dust and particulates being blown into the air (Nuyttens et al., 2013). In fact, the majority of the neonicotinoid applied does not end up in the target plant. Up to 95% ends up in the surrounding environment, being either blown away or washed into soil and water sources, having devastating effects for the ecosystem (Sur and Stork, 2003; Bonmatin et al., 2015) and often ending up in the pollen and nectar of wild, untreated plants (David et al., 2016). Although broken down fairly quickly by UV exposure, in the soil, neonicotinoids can have a half-life of over 3 years and many of their metabolites are also toxic (Bonmatin et al., 2015), leading to ongoing exposure long after their initial use. One study found that neonicotinoid concentrations in the soil of farmland annually planted with seed treated wheat increased by approximately 10 μg/L every year (Goulson, 2013).

## The Sub-Lethal Effects of Neonicotinoids on Beneficial Insects

The importance of nAChR receptor signaling in the insect brain results in the widespread lethal and sublethal effects of the drugs. The acute lethal dose of neonicotinoids can be remarkably low (e.g., 0.018 μg per honeybee for imidacloprid, Iwasa et al., 2004), and so insects can experience lethal doses of neonicotinoids in the field, particularly during spraying or sowing of treated crops. However, even at lower sub-lethal doses commonly found in nectar, e.g., 1–51 μg/L (Goulson, 2013) insects can experience a variety of behavioral disruptions.

Studies looking at the effects of field-realistic doses of neonicotinoids in honeybees and bumblebees have found that they can disrupt behaviors including learning and memory (Aliouane et al., 2009; Samuelson et al., 2016), locomotion (Aliouane et al., 2009), foraging success (Henry et al., 2012), foraging motivation (Lamsa et al., 2018), grooming behavior (Morfin et al., 2019), circadian rhythms and sleep (Tackenberg et al., 2020; Tasman et al., 2020). These behavioral effects at the individual level appear to culminate in reduced reproductive success at the colony level. Neonicotinoid exposure has been shown to lead to slower colony growth in bumblebees (Whitehorn et al., 2012). Neonicotinoids also appear to directly affect the fertility of the reproductive castes, reducing the viability of sperm in male honeybees (Straub et al., 2016) and reducing the fecundity of queens (Wu-Smart and Spivak, 2016). Neonicotinoids can also affect brain and neuronal development. In honeybees, neonicotinoid exposure has also been shown to increase apoptosis and mitochondrial dysfunction (Yan-Yan et al., 2014) of neurons and to cause a decrease in mushroom body (the memory center of the insect brain) synaptic density (Peng and Yang, 2016). This highlights how discrete subcellular effects can impact on important structures like the mushroom bodies that control a range of complex behaviors in insects including sleep (Eichler et al., 2017; Helfrich-Forster, 2018). In stingless bees, larval neonicotinoid exposure can reduce mushroom body mass by up to 36% in the resulting adult bee (Tomé et al., 2012). An important and emerging new sub-lethal effect of neonicotinoids is on bumblebee and honeybee circadian rhythms and sleep with similar effects demonstrated using *Drosophila* to allow mechanistic experiments, discussed later in this review (Tackenberg et al., 2020; Tasman et al., 2020, 2021).

It has previously been suggested that these effects are unlikely to occur in the field because insects have a choice of food and will avoid plants treated with neonicotinoids. However, work using bumblebees has since shown that neonicotinoids may be attractive rather than aversive to insects (Kessler et al., 2015). Additionally, although most studies focus on the effects of exposure to individual neonicotinoids, in the field insects often experience combined exposure to multiple pesticides (Botías et al., 2015), alongside environmental stressors such as pests and disease. Thus, disruptive effects observed in controlled, single exposure studies using field-relevant doses may impact more severely in the field (Gill et al., 2012; Goulson et al., 2015; Morfin et al., 2019; Straub et al., 2019).

## The Eu Ban of Neonicotinoids and Their Continued Global Use

In 2012 the EU initially approved five neonicotinoids: imidacloprid, clothianidin, thiamethoxam, thiacloprid and acetamiprid for agricultural use. However, due to the mounting evidence of the potential harm that these insecticides posed to beneficial insects, in 2013, the EU placed a moratorium on the use of imidacloprid, clothianidin and thiamethoxam, in order to severely restrict their use in the field and give time to scientists to determine their safety (European Food Safety Authority, 2011). In 2018, based on the accumulated safety data, it was voted that this ban should become permanent and also be extended to cover all field crops. Initially thiacloprid was not held within this ban, as it was shown to be less lethal to honeybees (Blacquiere et al., 2012). This difference in toxicity was likely to be due to the distinct chemical structure of the different classes of neonicotinoids; with imidacloprid, clothianidin and thiamethoxam all containing an N-nitro group, which makes them more reactive, while thiacloprid and acetamiprid contain a cyano-group (Iwasa et al., 2004). Furthermore the differential toxicity of neonicotinoids can be pharmacokinetic in origin, for instance a cytochrome P450 enzyme has been identified in honeybees and the solitary bee *Osmia bicornis* that can efficiently metabolize thiacloprid but has little efficacy against imidacloprid or the other N-nitro group neonicotinoids (Manjon et al., 2018; Beadle et al., 2019). However, due to mounting evidence of sub-lethal effects, in January 2020 thiacloprid was also added to the list of banned neonicotinoids (European Food Safety Authority, 2020), leaving acetamiprid as the only approved neonicotinoid. The approval of acetamiprid has already been renewed until 2033 due to ‘low risk to bees,’ although France has already banned its use based on its potential sub-lethal effects (French Government, 2017).

Despite the moratorium and resultant ban, there are still many routes for exposure for beneficial insects in the EU. Neonicotinoids can be used in greenhouses and gardens and are known to persist in the environment, so residues may still be in the environment from before the ban. For instance, one study compared the exposure of bumblebees to neonicotinoids in the UK the year before and the year after the moratorium of 2014 (Nicholls et al., 2018). They found that there was no significant reduction in the residue concentrations of banned neonicotinoids in nectar collected from bumblebee colonies for either rural or peri-urban environments but that there was a significant increase in thiacloprid concentrations after the 2014 moratorium. Another study in the UK found that over 20% of honey samples collected a year after the 2014 moratorium contained banned neonicotinoids (Woodcock et al., 2018). The highest concentrations of neonicotinoids were found in honey produced near oil seed rape *Brassica napus* plantations and during the flowering season, suggesting that despite not having been treated, these crops contained neonicotinoids due to ongoing environmental contamination.

Globally, neonicotinoids are still the most widely used pesticides, with registered use in 120 countries, and with precedent for revocation of pesticide bans. In 2014, the US banned the use of neonicotinoids in wildlife refuges, but this ban was overturned in 2018 by the Trump administration. In the United Kingdom numerous exemptions to the ban have been granted allowing the use of neonicotinoids on oil seed rape and carrot seeds (Casida, 2018). In addition to the continued use of neonicotinoids, the nAChR as a target site is still proving a fertile ground for the development of new brands of pesticides, with many analogous pesticides being currently synthesized and tested that essentially utilize the same mechanism of action (Kayser et al., 2004), but with a slightly distinct chemical structure that can therefore potentially circumvent legistation. Spinosad, another agonist of insect nAChRs (Salgado and Saar, 2004; Watson et al., 2010), is authorized for use in the EU and has even been approved for use on organic crops. This is despite evidence that the toxicity of spinosad to honeybees may be similar to that of imidacloprid (Challa et al., 2019; Christen et al., 2019). The mechanism of action and resistance to spinosad is being studied using whole genome sequencing of different *D. melanogaster* and *D. suzukii* strains that show different resistance levels, the polymorphisms associated with resistance are then edited into the genome using CRISPR mediated mutagenesis, with mutations in the α6 nAChR subunit being found to mediate resistance to spinosad (Zimmer et al., 2016; Mishra et al., 2018; Mendonca et al., 2019). This makes understanding the full suite of sub-lethal effects and the modes of action of neonicotinoids vital, as well as developing models for rapid identification of such effects caused by current and future pesticides.

## The Properties and Subunits of the Insect Nicotinic Acetylcholine Receptor

The nAChRs are the target of neonicotinoids and other pesticides and are the most abundant class of receptors in the insect brain (Dupuis et al., 2012). They show a high degree of conservation between *D. melanogaster* and other insects such as the honeybee, bumble bee, mosquito (*Anopheles gambiae*), the silk moth (*Bombyx mori*) and the red flour beetle (*Tribolium castaneum*) (Jones and Sattelle, 2010). The insect nAChR is a pentameric ligand-gated ion channel, composed of five nAChR subunits, forming a complex with an ion channel pore in the center (Dupuis et al., 2012). When open, this channel allows the movement of Na^+^, K^+^ and often Ca^2+^ across the membrane, allowing rapid changes in membrane potential. The insect nAChR subunits are divided into α and β types, and assemble to produce either α subunit homomeric nAChRs, α subunit heteromeric nAChRs or α and β subunit heteromeric nAChRs. ACh and agonists like nicotine and neonicotinoids bind to α subunits resulting in pore opening and depolarization (Dupuis et al., 2012). The subunit composition of the nAChR dictates its functional and pharmacological properties, producing a diverse number of nAChR subtypes. *D. melanogaster* was the first insect in which all nAChR subunits were characterized (Jones and Sattelle, 2010). There are ten subunits in *D. melanogaster*, Dα1-7 and Dβ1-3 (Dupuis et al., 2012). Subsequently nAChR subunit in other insect species have been grouped with respect to their homology to the *D. melanogaster* subunits. It appears that the nAChR subunits of most insects (including honeybees) can be placed into seven highly homologous groups, each with over 60% amino acid identity (Jones and Sattelle, 2010). Each insect also has distinct nAChR subunits, such as the Dβ3 subunit in *D. melanogaster* (Table 1). It will be interesting to discover the function of these species-specific subunits, the characterization of which may be instructive in developing truly species selective and hence safe insecticides. The occurrence of nAChRs in different brain regions also seems well conserved, with both *D. melanogaster* and *A. mellifera* having nAChRs in at least the optical lobe and tubercle, which process visual information, the subesophageal ganglion, involved in movement of the head, the antennal lobes which process olfactory information, and the mushroom bodies, which are involved in memory formation and sleep (Dudai and Amsterdam, 1977; Morley et al., 1979; Kreissl and Bicker, 1989; Jonas et al., 1990; Scheidler et al., 1990; Schuster et al., 1993; Hess et al., 1994; Thany et al., 2003; Wüstenberg and Grünewald, 2004; Thany et al., 2005; Barbara et al., 2008; Barnstedt et al., 2016).

**TABLE 1:** Comparison of genetic and molecular factors in *D. melanogaster* and bee species, to assess the usefulness of *D. melanogaster* as an insect model for pollinators.

	**Occurrence or appearance in *D. melanogaster***	**Occurrence or appearance in bee species**
Nicotinic acetylcholine receptors	There are 10 nAChR subunits: Dα1-7 and Dβ1-3.	There are 11 nAChR subunits: Amelα1-9 and Amelβ1-2. Of these, all except Amelα9 and Amelβ2 have over 60% identity with the equivalent *D. melanogaster* subunit. In both species, nAChRs are expressed in equivalent brain regions.
Cytochrome P450s	85 cytochrome P450 detoxifying genes identified.	Only 46 cytochrome P450 genes have been identified in *A. mellifera*, 44 in the bumblebee *B. huntii* and 52 in the leafcutter bee *Megachile rotundata.*
Genome	Published in 2000, exceptionally well annotated and accessible through https://flybase.org/with links to publicly funded stock centers that distribute fly mutants and reagents.	*A. mellifera* genome was published in 2006, followed by the bumblebees *B. terrestris* and *B. impatiens* in 2015.
Developmental genes	308 developmental genes highly conserved between *D. melanogaster* and *A. mellifera.*	308 developmental genes highly conserved between *D. melanogaster* and *A. mellifera.*
Neurotransmitters and Receptor genes	44 GCPRs, 21 biogenic amine receptors and 18 nuclear receptors in *D. melanogaster*	35 of 44 GCPRs, 19 of 21 biogenic amine receptors and all 18 nuclear receptors in *D. melanogaster* have close orthologs in *A. mellifera.*

Therefore gaining knowledge of the subunit composition and functional role of the different nAChR subtypes in distinct brain regions and species is an important area of research. However, this has been hampered by the difficulty in reconstituting functional heterologous insect nAChRs, with current studies reliant on artificial chimeric receptors formed by invertebrate and vertebrate subunits, in addition to a limited amount of data from nAChR subunit expression, co-localization and immunoprecipitation experiments (Jones and Sattelle, 2010). Based on this information it is proposed that there are three likely nAChR hetero-multimers; one involving at least Dβ1 and Dβ2 and an α subunit, one containing at least Dα1, Dα2, and Dβ2 and one with at least Dβ1 and Dα3 (Chamaon et al., 2002). Whilst we know that only some nAChR subtypes are susceptible to neonicotinoids (Moffat et al., 2016), the composition of these subtypes has not been fully determined. However, exploration of neonicotinoid resistance using *D. melanogaster* as a model has identified some nAChR subunits which appear to be involved in susceptibility, such as Dα1, Dβ1, and Dβ2 (Perry et al., 2012; Homem et al., 2020), in contrast α6 nAChR subunit is important for spinosad’s mode of action and resistance (Zimmer et al., 2016; Mishra et al., 2018; Mendonca et al., 2019).

## The Use of *D. melanogaster* as the Insect Model Organism

*Drosophila* has been intensively studied for over a century and is perhaps the most genetically tractable of all the model organisms with work conducted in flies being acknowledged by six Nobel prizes (Hales et al., 2015). The *D. melanogaster* genome was published in 2000 (Adams et al., 2000), while the honeybee genome was released in 2006 (Weinstock et al., 2006), followed by the bumblebees *B. terrestris* and *B. impatiens* in 2015 (Sadd et al., 2015). Due to the genome of *D. melanogaster* having been so intensively studied and annotated, genes tend to be identified in pollinators due to their close homology to those previously identified and characterized in *D. melanogaster*. This allows a comparison of the genes between species, providing information on how conserved many of the gene groups are. Genome and phylogenetic comparison of the nAChR gene family between insects provides insight into their physiological, pharmacological or functional roles suggesting how best to model them in *D. melanogaster*, while species specific differences especially between pest and pollinator nAChR likewise provide the promise of developing future insecticides with selective toxicity and no effects on non-target species.

Generally, there appears to be a high level of genetic conservation between *D. melanogaster* and pollinators (Table 1), with good evidence for molecular conservation with *A. mellifera* genes, perhaps because after flies the honeybee genome has been most widely annotated. For example the *sex-lethal* (*sxl*), *doublesex* (*dsx*), and *intersex* (*ix*) genes, which are involved in somatic sex determination as well as sex-specific brain and genital development, show 68, 51, and 43% nucleotide identity between *D. melanogaster* and *A. mellifera* (Cristino et al., 2006). Despite the very different sex determination processes between the two species. It should be noted that these genes are not involved in sex selection in all Drosophilidae species. This genetic conservation between *A. mellifera* and *D. melanogaster* appears to be true for most developmental genes; with 308 important developmental genes in *D. melanogaster* found to be highly conserved in *A. mellifera* (Dearden et al., 2006).

As well as developmental genes, many neurotransmitters and receptors appear to be well conserved between fruit flies and honeybees. Most G-protein-coupled receptor genes, a very large gene family in most organisms, important in for instance olfaction, were originally identified in *D. melanogaster* and appear to have close orthologs in the honeybee. Of the 44 identified in *D. melanogaster*, 35 had a close ortholog in *A. mellifera*, with the greater number in *D. melanogaster* likely being down to duplications in recent evolutionary time (Hauser et al., 2006). Honeybees were also shown to have 19 of the 21 biogenic amine receptors identified in *D. melanogaster* important for neuronal communication (Hauser et al., 2006). Additionally, all of the 18 nuclear receptors found in *D. melanogaster* have orthologs in the honeybee and are important for maturation and development (Velarde et al., 2006).

## The Versatile Genetic Toolbox of *D. melanogaster*

*D. melanogaster* offers a number of powerful tools for dissecting gene function and behavior including the *GAL4/UAS* and analogous promoter system for targeted spatiotemporal expression of transgenes, a genome wide library of *RNAi* transgenes allowing targeted knockdown of any gene and CRISPR-Cas9 library allowing double stranded DNA breakpoints in any gene to carry out genome editing (Duffy, 2002; Gratz et al., 2015; Heigwer et al., 2018). The *GAL4*-*UAS* system is one of many promoter systems that allow tissue-specific gene expression. It is binary, consisting of one library of flies that express the yeast Gal4 transcription factor in different spatiotemporal patterns. The second part of the system consists of collections of flies which contain the *Gal4* responsive upstream activator sequence (*UAS*) prior to any transgene of interest. By themselves, *Gal4* or *UAS* in a fly genome are inert as the yeast system has no endogenous targets or equivalents in flies, making the *Gal4* or *UAS* alone stock essentially wildtype. However, when you cross your chosen *Gal4* line to a UAS line of interest, then in the heterozygote offspring you have both parts of the system, allowing misexpression of the transgene wherever the Gal4 promoter drives expression. Libraries of *GAL4* lines have been created with *GAL4* expression in specific tissues, brain regions or even a small number of neurons due to the use of intersection promoter systems. Therefore when crossed together you can mis-express any gene wherever you want to. In addition there are range of inducible promoters that allow you to express a gene in a particular pattern during a particular time window, perhaps during a particular phase of development or just in adulthood before behavioral testing. The Gal4/UAS system typically allows 3- to 10-fold overexpression of a gene depending on the choice of line and experimental condition (Hodge, 2009; Hales et al., 2015; Senturk and Bellen, 2018). Conversely expression of a transgene expressing a double-stranded RNA (*dsRNA*) designed to be complementary to any specific endogenous target gene, allows targeted knockdown of that gene, in the Gal4 pattern of choice. The dsRNA is cleaved by an enzyme called dicer (which can also be overexpressed with Gal4/UAS to increase knockdown) into two single strands, the guide strand then binds to the complementary endogenous mRNA, inducing cleavage that results in post-transcriptional silencing of the target gene. Again, depending on the choice of *UAS-RNAi* line and various experimental options, this allows typically 10–90% knockdown of the target gene in a particular tissue or neuronal type depending on the Gal4 promoter which was chosen (Hu et al., 2013; Hales et al., 2015; Senturk and Bellen, 2018).

The CRISPR-Cas9 system is a genome editing tool, allowing both the double stranded cleavage of DNA and subsequent insertion of strands of DNA into that break. This system is derived from a bacterial immune response pathway involving a Cas9 complex containing the guide RNA which is complimentary and specific to the genomic DNA sequence that needs to be cut. This guide RNA recruits the Cas9 enzyme to this site where it cuts the DNA. This produces a double strand break, causing a functional knockout of the gene or allowing the insertion of an alternate gene (e.g., GFP) or a mutant version of the endogenous gene. Alternatively, the cutting domains of Cas9 can be deactivated and other enzymes fused to it that allow alteration of the nucleotide sequence of the DNA, allowing fine scale gene editing. Other uses include fusing of transcriptional machinery or CRAB domains to cause over-expression or silencing of the gene. These tools provide a powerful means of investigating the role of genes *in vivo*. CRISPR-Cas9 has revolutionized biology, as it can be used in any species and makes creating transgenes an order of magnitude easier. *RNAi* and CRISPR techniques are being introduced into other insects, thereby facilitating translation of *D. melanogaster* findings, resources and tools quickly to a diverse range of ecologically important insects with the potential of making them all model organisms of the future (Matthews and Vosshall, 2020). Already these tools are starting to be used in pollinators such as honeybees (Hu et al., 2019) and a range of butterflies (Perry et al., 2016; Concha et al., 2019). This shows the benefit of a parallel approach; developing techniques quickly and efficiently in the genetically and experimentally tractable *D. melanogaster* then transferring the knowledge and tools gained to other species such as pollinators, allowing evolutionary and ecological comparisons. This approach is also set to give us a more holistic and deeper understanding of the biology of life, which has largely been gained through studying the gut bacteria (*Escherichia coli*), baker’s and fission yeasts (*Saccharomyces cerevisiae* and *Schizosaccharomyces pombe*), thale cress weed (*Arabidopsis thaliana*), nematode (*Caenorhabditis elegans*), fruit fly (*D. melanogaster*), zebrafish (*Danio rerio*), mouse (*Mus musculus*), and rat (*Rattus norvegicus*).

## Modeling Eusocial Behaviors and the Limits of the Fly Model

The sharing of the basic building blocks for development and communication described in the last sections mean that *D. melanogaster* can be used to model even seemingly quite derived or specialized behaviors seen in pollinators. For example, a number of studies have focused on modeling aspects of honeybee queen development and pheromones using *D. melanogaster*. These often successfully replicate responses that are highly linked to the eusocial nature of honeybees, a very specialized behavior that is not observed in *D. melanogaster.* Eusocial behavior consists of cooperative brood rearing including looking after the offspring of other individuals, with division of labor splitting groups or castes into those who do and do not reproduce and with overlapping generations within a colony (Camiletti et al., 2013; Shorter et al., 2015; Croft et al., 2017).

Certain substances, such as royal jelly, cause these differences to occur for instance by being fed to honeybee queens as larvae resulting in them becoming differentiated from their sister worker bees by causing the queen to grow larger, live longer and develop fully functional ovaries (Kamakura, 2011). Strikingly comparable effects can be induced in *D. melanogaster* females when fed royal jelly resulting in increased viability, elongated development time, increases in thorax length and weight (Shorter et al., 2015). The effects of queen mandibular pheromone (QMP), a pheromone that mediates eusocial behavior in honeybees, has also been tested on male and female *D. melanogaster*. In male honeybees, QMP causes sexual attention, but in female workers it causes increased foraging and brood care efforts and prevents ovary development (Butler and Fairey, 1964; Hoover et al., 2003; Higo et al., 2012). QMP also stifles ovary development in female *D. melanogaster*, causing them to have smaller ovaries, lay fewer eggs and produce less viable offspring (Camiletti et al., 2013). Male *D. melanogaster* are attracted to honeybee QMP, showing a preference for it in a T-maze set up. Furthermore, exposure to QMP increases male fly’s sexual attentiveness when paired with female conspecifics (Croft et al., 2017). The capacity to replicate honeybee behaviors in *D. melanogaster* reinforces their evolutionary relatedness and makes it possible to explore the genetic pathways underlying these honeybee behavioral changes in flies. For example, identification of genes that change when virgin female *D. melanogaster* are exposed to QMP identified the olfactory co-receptor ORCO, alongside a number of olfactory receptors, that mediate detection of QMP and its effects on fecundity (Camiletti et al., 2016). *Orco* is a gene that has previously been implicated in eusocial behaviors, with CRISPR having been used to knock out the gene in ants causing them to fail to follow pheromone trails or congregate with other ants (Trible et al., 2017).

Despite these widespread similarities, it is important to recognize that *D. melanogaster* and pollinators occupy different niches, resulting in genetic divergence that may impact the consumption and metabolism of neonicotinoids and thus their sub-lethal effects. For instance, comparison of the genes for enzymes involved in carbohydrate and lipid metabolism showed that, whilst there tended to be a high level of conservation for these genes between the two species, there were a number of striking changes and these tended to be in genes involved in the metabolism of glucose (Kunieda et al., 2006). This is likely to suggest functional differences in glycolysis between the two species, perhaps due to the need for a very high carbohydrate diet in *A. mellifera* required to perform energetically costly behaviors such as long foraging flights and heat generation in the colony. *A. mellifera* also possess just a third of the genes implicated in immunity compared to *D. melanogaster*, suggesting that the colony environment may protect them from disease or that their lifestyle leads to fewer possibilities for infection (Evans et al., 2006). Similarly, the genes identified as participating in the antioxidant system in *D. melanogaster* all have orthologs in honeybees, but there is a difference in the number of paralogs for each gene in the two species. Many antioxidant genes are duplicated and expanded in *D. melanogaster* but not in the honeybee, suggesting that bees may be susceptible to stress, hence their greater sensitivity to neonicotinoids than pests (Corona and Robinson, 2006). Additionally, *D. melanogaster* have markedly more cytochrome P450 genes, which encode the detoxification enzymes that metabolize neonicotinoids. In *D. melanogaster* there are 85 of these P450 genes, compared to 46 in honeybees, 44 in the bumblebee *B. huntii* and 52 in the leafcutter bee *Megachile rotundata* (Berenbaum and Johnson, 2015) (Table 1). Further to this, functional expression of *D. melanogaster*, honeybee and *B. terrestris* nAChRs in *Xenopus* oocytes showed that honeybee and bumblebee receptors appear to be more sensitive to neonicotinoids (Ihara et al., 2020). With these limitations in mind, there are still many advantages and successful examples of *D. melanogaster* being utilized to model and explore the mechanism of action for sub-lethal effects of neonicotinoids described in the next section. Similarly, by using a parallel comparative approach one learns more about the biology, evolution and response to neonicotinoids of the different species.

The following section reviews the different behavioral and physiological effects of neonicotinoids in different pollinators with respect to *D. melanogaster* including underlying mechanisms of action and is summarized in Table 2.

**TABLE 2:** Summary of effects of neonicotinoids on *D. melanogaster* physiology and their ability to models the effects on bee species.

	**Effect of neonicotinoids on *D. melanogaster***	**Relevance of *D. melanogaster* for understanding impact of neonicotinoids on bee species**
Lethality	Numerous studies have demonstrated that neonicotinoids have effects on *D. melanogaster* longevity and survival, and these effects can vary according to sex.	Although *D. melanogaster* have a higher tolerance to neonicotinoids than bee species, sublethal effects appear to occur in the same range of field relevant concentrations as in bee species, and the relative toxicity of each drug appears to be the same i.e., clothianidin > thiamethoxam > imidacloprid > thiacloprid.
Susceptibility and resistance	Mechanisms of resistance to the effects of neonicotinoids have been characterized in *D. melanogaster*, such as mutations in the cytochrome P450’s and nAChR subunits.	The key mechanisms of resistance identified in *D. melanogaster*, explain the susceptibility of bee species to neonicotinoids. Bees have far fewer cytochrome P450’s than *D. melanogaster* and have nAChRs that appear more neonicotinoid sensitive.
Reproduction and development	Both behavioral and physiological effects of neonicotinoids have been characterized in the reproductive system of *D. melanogaster.*	Neonicotinoids cause a range of effects on mating behavior and success in bees, many of which are closely mirrored in *D. melanogaster*, i.e., reduced egg laying and viability. This suggests potential for *D. melanogaster* to determine the underlying mechanism.
Locomotion and motor function	Differing neonicotinoids may cause hyperactivity or reduction of locomotion in *D. melanogaster* and have been shown to reduce climbing behavior with a role of Dβ1 mediating these effects.	As in *D. melanogaster*, neonicotinoids cause both hyperactivity and inactivity, dependant on drug and dose, whilst also reducing complex motor skills such as climbing.
Immune system	Neonicotinoids affect the immune system of *D. melanogaster* by actions, which have been physiologically characterized.	Although there are differences between the immune systems of *D. melanogaster* and bee species, a comparative approach has demonstrated that some analogous components of these immune systems are affected similarly by neonicotinoids.
Olfaction and memory	Neonicotinoids induce learning and memory deficits in *D. melanogaster.* Mushroom body Kenyon cell synapses are nicotinic with Dα1 and Dβ2 mediating the effects of neonicotinoids on learning and memory.	In bees, neonicotinoids have also been shown to reduce learning, and to influence mushroom body size and connectivity.
Circadian rhythmicity	Neonicotinoids disrupt the circadian clock of *D. melanogaster.* Knockdowns of Dα1 and Dβ2 in clock cells mimic the effects of neonicotinoids on circadian behaviors and clock neuron morphology. Neonicotinoids had no further effect on circadian rhythms of flies with clock mediated Dα1 and Dβ2 knockdown, suggesting these receptors do indeed mediate the effect of the drugs on this behavior.	Neonicotinoids also disrupt the circadian clock in bees, in a similar way to *D. melanogaste*r and Dα1 and Dβ2 are likely involved as they are well conserved between these insects. It was the identification of these effects in *D. melanogaster* that led to circadian rhythmicity being tested in bees.
Sleep	Neonicotinoids can lead to fragmented and reduced sleep in *D. melanogaster* and is mediated by Dα1 and Dβ2 receptor function in the clock	Neonicotinoids also effect the quantity and timing of sleep achieved in bee species.
Other neuronal and metabolic functions	Other mechanistic effects of neonicotinoids on *D. melanogaster* have been reported – see the main text for details.	It would be interesting to test if these mechanisms are also conserved in bees

## *D. melanogaster* as a Model for the Effects of Neonicotinoids On: Lethality

Neonicotinoids have proven lethal to pollinators at very low doses. In honeybees, an oral dose of just 0.003 μg per bee of clothianidin killed 50 percent of bees within 48 h [Lethal Dose_50__%_ (LD_50_) 48 h]. For thiamethoxam the oral LD_50_ 48 h per bee was 0.005 μg, for imidacloprid 0.03 μg and for thiacloprid it was 17.3 μg (Blacquiere et al., 2012). Acute contact *via* spraying of imidacloprid solution proved lethal to 50% of the population [Lethal Concentration_50__%_ (LC_50_)] in 48 h at a concentration of 7 mg/L for the orchard mason bee *O. lignaria*, 17 mg/L for the leaf cutting bee *M. rotundata* and 322 mg/L for the bumblebee *B. impatiens* (Scott-Dupree et al., 2009). Contact with concentrations of 7.5–100 μg/L imidacloprid, such as solitary bees may experience in the soil during ground-nesting, have also been shown to affect longevity in *O. lignaria* and *M. rotundata* (Anderson and Harmon-Threatt, 2019). This introduces an important concept, that of the field-relevant dose or concentration of neonicotinoid, defined as the amount of a neonicotinoid an insect might actually get exposed to in the environment. Field-relevant concentrations of neonicotinoids are 1–51 μg/L for seed treated crops and 61–127 μg/L for sprayed crops (Wood and Goulson, 2017). Chronic exposure to these doses is not necessarily lethal; both honeybees and *B. terrestris* showed no reduction in longevity when fed between 0.1 and 100 μg/L imidacloprid daily, although the study period was around 30 days for bumblebees and 6 days for honeybees (Cresswell et al., 2012). Similarly, honeybees fed 4 μg/L of thiamethoxam and 2 μg/L of clothianidin showed no change in longevity (Straub et al., 2019). However, these low, field-realistic doses can have severe sub-lethal effects. Concentrations as low as 1 μg/L [equivalent to 1 part per billion (ppb)] have sub-lethal effects such as reducing foraging motivation in the bumblebee *B. terrestris* (Lamsa et al., 2018) with similar but slightly higher concentrations decreasing honeybee locomotion (Williamson et al., 2014). 1–100 μg/L imidacloprid and clothianidin disrupted butterfly development and behavior (Basley and Goulson, 2018; Whitehorn et al., 2018).

The lethality and longevity effects of neonicotinoids have also been tested in *D. melanogaster.* The LC_50_ 18 h for imidacloprid is 333 mg/L for males, whilst females appear to be less susceptible, with an LC_50_ 18 h of > 791 mg/L (Charpentier et al., 2014). Thiamethoxam was found to have an LC_50_ over 24 h of 3.13 μg/L for adult *D. melanogaster* (Li et al., 2020). These results suggest that neonicotinoids may be less lethal to *D. melanogaster* than to pollinators, with an LC_50_ for flies much higher than for the species mentioned above. This may be due to factors such as the greater number of cytochromes P450s that *D. melanogaster* possess, or because the nAChRs of honeybees and bumblebees appear to be more neonicotinoid-sensitive than those of *D. melanogaster* (Berenbaum and Johnson, 2015; Ihara et al., 2020). One study comparing the effects of field-relevant doses of neonicotinoids on longevity in *D. melanogaster*, found that chronic oral exposure to 10 μg/L of neonicotinoid reduced lifespan (Tasman et al., 2021). Clothianidin had the greatest effect, followed by imidacloprid and thiamethoxam and lastly by thiacloprid. This is consistent with concentrations seen in the field and recapitulates the effects of the different neonicotinoids on pollinators, validating the use of *Drosophila* as a lab-based model for studying the effects of neonicotinoids on pollinators (Ellis et al., 2017; Stanley and Raine, 2017; Wood and Goulson, 2017; Woodcock et al., 2017; Basley and Goulson, 2018; Crall et al., 2018; Wintermantel et al., 2020). The range of pollinator studies summarized above show how comparisons between different neonicotinoids can be difficult to make due to the wide range of concentrations and methods of exposure used and their relevance to actual field-relevant concentrations. However *D. melanogaster* has the potential to allow rapid high throughput screening of multiple neonicotinoids at a range of concentrations, allowing comparative studies of the lethal and sub-lethal effects they cause, allowing the efficacy and adverse effects of each to be assessed.

## Susceptibility and Resistance

Neonicotinoid susceptibility or resistance is mainly conferred by nAChR subunit mutations (Perry et al., 2008; Somers et al., 2017), such as the nAChR β1 subunit R81T mutation which results in aphids being more resistant and the equivalent genomically engineered *D. melanogaster* being 30 times more resistant to imidacloprid (Homem et al., 2020). Likewise upregulation of neonicotinoid metabolizing enzymes such as cytochrome P450s is another widely reported mechanism of resistance, replicated in *Drosophila* mutants that overexpress individual P450 genes (Kalajdzic et al., 2012; Denecke et al., 2017). However, coming back to the question of fitness consequences, others have argued that because fly nAChR gene null mutations have pleiotrophic effects, especially in terms of behavioral defects, that there must be fitness consequences to such resistance mutations (Somers et al., 2017, 2018). Clearly such complex genotype and phenotype interactions are well suited for genetic dissection using *D. melanogaster*, especially as now it is considered that resistance is not necessarily the result of a binary ON/OFF mechanism such as the loss of a functional drug binding site or ion channel subunit gene, but rather a graded process of loss of affinity, efficacy or changes in expression to less sensitive nAChR subunits, thermotolerance or mutations causing a behavioral change such as loss of avoidance (Casida and Durkin, 2013; Kessler et al., 2015; Benzidane et al., 2017; Fournier-Level et al., 2019; Matsuda et al., 2020).

One tool unique to flies useful for addressing such questions is the *D. melanogaster* genetic reference panel (DGRP), which consists of 200 inbred lines derived from a single population of wild-caught flies. Each line has been whole-genome sequenced and phenotypically characterized for multiple traits (Mackay et al., 2012). These include imidacloprid susceptibility, which varies between the strains based on variations in neuronal genes, e.g., *nAChRβ1 R81T* and *nAChRα6* deletion flies (Homem et al., 2020) and cytochrome P450s (Denecke et al., 2017), and imidacloprid resistance which can be conferred by overexpression of metabolizing enzymes (e.g., cytochrome P450s) (Kalajdzic et al., 2012; Denecke et al., 2017). There were differences amongst these inbred *D. melanogaster* lines in lethal effects and resistance to imidacloprid in larvae compared to adults in these strains, and adults did not fully recover after just larval imidacloprid exposure (Young et al., 2019). *D. melanogaster* has a number of congeneric species including *D. suzukii*, an important invasive species and major pest of soft fruit in the United Kingdom (Mishra et al., 2018; Orsted and Orsted, 2018), which has been characterized for neonicotinoid lethal effects and its genomic response to the insecticides (Mishra et al., 2018). Further studies using DGRP, Genome Wide Association Studies and more conventional forward genetic screens offer huge potential to reveal more subtle and rare gene mutations and mechanisms underlying neonicotinoid susceptibility and resistance.

## Reproduction and Development

Neonicotinoid exposure has been shown to affect reproduction and offspring survival. In honeybees, thiamethoxam (4.5 μg/L) and clothianidin (1.5 μg/L) can reduce the quantity of living sperm by 39% in male honeybees (Straub et al., 2016). Similarly, honeybee queens exposed to thiamethoxam (4 μg/L) and clothianidin (1 μg/L) were found to have 20% less sperm stored in their spermatheca and this sperm was 9% less viable than in control queens, despite having had a similar number and duration of mating flights (Williams et al., 2015). Exposed queens were found to lay fewer eggs and a smaller percentage of these eggs successfully developed into adults. Larvae also require longer to develop when exposed to imidacloprid (5 μg/kg) (Decourtye et al., 2005). This effect has been seen in *O. lignaria* at a dose of 30–300 μg/kg (Abbott et al., 2008). Exposure to imidacloprid (2.5 μg/L) or thiamethoxam (2.9 μg/L) both appear to severely reduce the number of brood cells in *B. terrestris* colonies, by 46 and 70% respectively, whilst clothianidin (2.5 μg/L) does not appear to have an effect (Moffat et al., 2016). Exposure of *B. terrestris* micro-colonies to imidacloprid in nectar (10 μg/L) and in pollen (6 μg/kg) also caused a reduction in the number of brood (Tasei et al., 2000). While imidacloprid (5 μg/L) exposure can cause *B. terrestris* queens to become less active, delay nest initiation and produce less brood (Leza et al., 2018). Exposure to 6 μg/L of neonicotinoids has also been shown to disrupt bumblebee nest behavior (Whitehorn et al., 2012; Crall et al., 2018).

Likewise, imidacloprid, clothianidin, thiamethoxam and thiacloprid have all been shown to reduce fecundity, offspring viability and lifespan of *D. melanogaster* in a similar fashion (Charpentier et al., 2014; Li et al., 2020; Tasman et al., 2021). Exposure to imidacloprid (0.1–0.5 μg/L) caused an increase in mating, whilst higher concentrations (0.5–10 μg/L) caused a reduction in the number of offspring produced (Charpentier et al., 2014). Thiamethoxam was found to cause a dose-dependent decrease in egg laying, pupation, eclosion rate, and third-instar larval survival (Li et al., 2020). Another study found that imidacloprid (10 μg/L), clothianidin, thiamethoxam or thiacloprid (100 μg/L) all caused a reduction in the number of eggs that survived to adulthood (Tasman et al., 2021). Similarly, RNAi mediated knockdown of the Dα1 neonicotinoid-susceptible nAChR subunit in the nervous system (Perry et al., 2012) caused males to delay initiating courtship and copulation, suggesting that this subunit is normally involved in mating behavior and that neonicotinoids effect this behavior via Dα1 (Somers et al., 2017). Similarly, flies with a mutation in Dβ1, another neonicotinoid susceptible subunit also showed reduced longevity, fecundity and egg survival (Homem et al., 2020). Such *Drosophila* studies provide a valuable insight into the composition of neonicotinoid-susceptible nAChRs, where the individual nAChR subunits are expressed and what their individual roles are in insect behavior and neonicotinoid responses.

## Locomotion and Motor Function

Neonicotinoid exposure has been shown to affect basic locomotion and activity, as well as more complex motor co-ordination. In the stingless bee *Tetragonisca angustula*, imidacloprid (1.7 mg/L) or thiacloprid (0.28 mg/L) caused a reduction in locomotor activity, whilst thiamethoxam (0.28 mg/L) caused hyperactivity (Jacob et al., 2019). Imidacloprid also caused reduced locomotor activity in the bumblebee *B. terrestris* (Cresswell et al., 2014) and the stingless bee *Melipona quadrifasciata* (Tomé et al., 2012) at doses of 125 μg/L and 28–112 ng/bee respectively. In honeybees, thiamethoxam (1.34 ng/bee) and clothianidin (1–2 ng/bee) have been shown to cause hyperactivity (Tosi and Nieh, 2017; Alkassab and Kirchner, 2018). A high dose of imidacloprid (2.5–20 ng/bee) has been shown to reduce locomotor activity, while a lower dose (1.35 ng/bee) increased locomotion (Lambin et al., 2001). In a study looking at honeybee flight, a single dose of thiamethoxam (1.34 ng/bee) caused hyperexcitation, but continued dosing over 1–2 days caused a reduction in flying ability (Tosi et al., 2017). This duality, with neonicotinoids capable of eliciting both locomotor hyperactivity and inactivity depending on dose and duration of exposure, occurs frequently in the literature and may be connected to initial agonist action of neonicotinoids opening nAChRs and exciting neurons and then perhaps prolonged or high doses causing depolarizing block, receptor desensitization and inactivation of neurons (Tan et al., 2007).

Despite often causing hyperactivity and increased locomotion, neonicotinoids also appear to reduce the ability of bees to perform more complex motor tasks. Exposure of honeybees to thiamethoxam (1.42–3.48 ng/bee) caused a reduced capacity to climb up a vertical surface toward light (Tosi and Nieh, 2017), while clothianidin (1–2 ng/bee) resulted in a loss of postural control and an increase in the proportion of time spent upside down (Alkassab and Kirchner, 2018). Similarly, imidacloprid, clothianidin (2.5 μg/L) or thiamethoxam (2.9 μg/L) caused loss of postural control and more time upside down (Williamson et al., 2014). Thiamethoxam also caused the bees to spend more time grooming. Conversely, clothianidin (0.9 μg/L) caused a reduction in grooming (Morfin et al., 2019). As well as affecting locomotor activity, neonicotinoids can affect foraging and in-nest activity. Exposure to either imidacloprid (0.15–6 ng/bee) or clothianidin (0.05–2 ng/bee) reduced foraging activity in honeybees (Schneider et al., 2012) and imidacloprid reduced foraging activity in *B. terrestris* at 10 μg/L (Gill and Raine, 2014; Tasman et al., 2020), and reduced in-nest activity at 6 μg/L (Crall et al., 2018). Neonicotinoid exposure also appears to change the expression of hundreds of genes in honeybee foragers, including genes which are involved in locomotion, such as titin (Mobley and Gegear, 2018). Likewise 6 μg/L neonicotinoid changed the whole head transcriptome of bumble bees with workers being more sensitive than queens and genes in being involved in mitochondrial function being particularly affected (Colgan et al., 2019).

In *D. melanogaster*, clothianidin (10–50 μg/L) and thiamethoxam (1 μg/L) caused hyperactivity, whilst imidacloprid (50 μg/L) caused reduced locomotor activity (Tasman et al., 2021). As seen for pollinators in the examples above, despite causing hyperactivity, clothianidin and thiacloprid (10 μg/L) reduced the ability of flies to carry out a more challenging motor task (climbing), as did imidacloprid. The originally non-banned thiacloprid on the other hand did not appear to affect locomotion or climbing (Tasman et al., 2021). Neonicotinoid susceptible Dβ1 subunit mutant flies displayed reduced larval crawling and adult climbing, again suggesting a role for this subunit in the effects of neonicotinoids on mobility (Homem et al., 2020).

## The Immune System

Despite the potential differences in immune function, *D. melanogaster* has still provided valuable insights into the effects of neonicotinoids on the immune system of bees. Neonicotinoids have been shown to reduce the immune response in honeybees and thus increase their vulnerability to pests (Di Prisco et al., 2013). Recent work in *D. melanogaster* has identified a potential pathway for this effect, showing that neonicotinoid exposure caused an increase in the transcription of a gene which inhibits NF-kB immune signaling, reducing antiviral defenses in the insect (Di Prisco et al., 2013). This gene called *Dmel/LRR*, was identified in *D. melanogaster* due to its high sequence similarity to the equivalent gene (*CLR16.2*) in humans. The gene’s role in the immune pathway was already well established in vertebrates and had been shown to be responsive to neonicotinoids in the mussel *Mytilus galloprovincialis* (Dondero et al., 2010), but had not previously been identified in insects. After exposure to clothianidin, *D. melanogaster* had reduced NF-kB activation and thus a reduced immune response when immunologically challenged. This was validated in bees through measuring the transcription levels of *Amel/LRR*, the honeybee ortholog of *Dmel*. After exposure to clothianidin, bees given an immune challenge showed up-regulation of *Amel/LRR*, suggesting that clothianidin also dampens the immune response in honeybees. This study clearly illustrates the value of a comparative approach, utilizing the tools available in *D. melanogaster* to identify sub-lethal effects and the mechanisms responsible for them and allowing these to then be validated in pollinators. Identifying the specific mechanisms through which neonicotinoids disrupt vital processes in insects such as immunity, reproduction and behavior allows us to build a more comprehensive understanding of its effects in the body, other potential off-target effects that may be occurring as well as the function of nAChRs in healthy insects. This should prove essential information for the design of safer and more effective insecticides for the future.

A number of other potential mechanisms of neonicotinoid effects on the invertebrate immune system have been identified using *D. melanogaster*. For instance, imidacloprid reduces phagocytosis by *D. melanogaster* hemocytes which are the invertebrate immune system cells (Walderdorff et al., 2019). Imidacloprid has also been shown to interfere with the dual oxidase (duox) pathway in *D. melanogaster*, reducing the production of hydrogen peroxide which helps control infection and maintain the gut microbiome (Chmiel et al., 2019). This resulted in an imidacloprid mediated increase in risk of infection and changes in the gut microbiome of *D. melanogaster.* These effects could be mitigated through the addition of probiotic *lactobacilli* (Daisley et al., 2017; Chmiel et al., 2019). It was also found that the neonicotinoid nitenpyram disrupted the honeybee gut microbiome (Zhu et al., 2020), whilst imidacloprid did not (Raymann et al., 2018). Likewise clothianidin did not affect the bumble bee microbiome (Wintermantel et al., 2018). The use of probiotics to boost honeybee immune response is a current topic of research and may mitigate against the effects of neonicotinoids.

## Olfaction and Memory

Neonicotinoid exposure can have dramatic effects on the memory of pollinators. Given the importance of memory for pollination and navigation, this is like to negatively impact the bee’s performance in the field and hence our food security. *B. terrestris*, when exposed to thiamethoxam (2.4 μg/L) showed a reduction in learning and short-term memory in an appetitive olfactory assay called the proboscis extension reflex (PER) task (Stanley et al., 2015), where a bee learns to extend its proboscis or feeding parts when an odor is given at the same time as a sugar reward. In *A. mellifera*, clothianidin (4 μg/L), thiamethoxam (1.9–2.9 μg/L) and imidacloprid (2.5–24 μg/L) all reduced this type of appetitive olfactory learning (Decourtye et al., 2004; Williamson and Wright, 2013; Wright et al., 2015; Piiroinen and Goulson, 2016; Mustard et al., 2020). In *A. mellifera jemenitica*, 0.1–1 ng/L imidacloprid caused a similar reduction in memory performance (Iqbal et al., 2019) with similar effects reported when the eastern honeybee *A. cerana* was exposed to 0.1–1 ng imidacloprid/bee (Tan et al., 2015). Imidacloprid (25.6 μg/L) also reduced aversive olfactory learning, reducing the sting extension response (SER) of *A. mellifera* to a simulated predator (Zhang and Nieh, 2015). However, imidacloprid (5–20 ng/bee) applied to the thorax of *A. mellifera* reduced its gustatory threshold, facilitating PER habituation (Lambin et al., 2001). Exposure of the solitary bee *O. cornuta* to clothianidin (0.76 ng/bee) interfered with the retrieval of navigational memory (Jin et al., 2015). Navigation and homing success were also reduced in *A. mellifera* by exposure to imidacloprid, clothianidin or thiacloprid (Fischer et al., 2014). In *B. terrestris*, thiamethoxam (0.091–2.5 ng/bee) reduced spatial working memory, tested using a radial arm maze (Samuelson et al., 2016). Furthermore, thiamethoxam (10 μg/L) also reduced the ability of *B. terrestris* to learn efficient flower handling whilst foraging (Stanley and Raine, 2016).

In addition to affecting behavior, neonicotinoids have also been shown to affect the neurons and brain structures involved in olfactory learning, likely underlying or contributing to the behavioral defects reported. Neonicotinoid exposure affects the development of the mushroom body, the brain region involved in memory formation in insects (Busto et al., 2010). Developmental exposure to imidacloprid (5 μg/L) in *B. terrestris* reduced mushroom body growth and caused learning deficits in adults (Smith et al., 2020). In *A. mellifera*, exposure to imidacloprid (1–500 μg/L) decreased the synaptic density of the mushroom bodies (Peng and Yang, 2016). Similarly, in the stingless bee *M. quadrifasciata* imidacloprid exposure during development resulted in a 36% reduction in the volume of the mushroom body of adult bees (Tomé et al., 2012). In *A. mellifera*, exposure to imidacloprid or clothianidin caused a depolarizing block of mushroom body neurons, inhibiting firing of action potentials and hence neuronal communication within the memory center (Palmer et al., 2013). Neonicotinoids also reduced honeybee antennal lobe intracellular Ca^2+^ responses resulting in sensory deficits (Andrione et al., 2016).

Neonicotinoids also induce learning and memory deficits in *D. melanogaster*, where 10 μg/L of imidacloprid, clothianidin or thiamethoxam reduced 1 h olfactory shock memory (Tasman et al., 2021). Additionally, two neonicotinoid-susceptible nAChR subunits, Dα1 and Dβ2 were knocked down *via* RNAi expression in the mushroom bodies. Either loss of function mutant had a similar reduction in 1 hr memory as neonicotinoid exposed flies, implicating mushroom body-expressed Dα1 and Dβ2 in memory and the effects of neonicotinoids on memory. The subunits Dα1 and Dα4-6 also appear to be involved in the olfactory response, as *RNAi*-mediated knockdown of any of these subunits in the mushroom body output neurons causes a change or even a reversal in response to olfactory stimuli and intracellular Ca^2+^ response, measured using Gal4 mediated expression of the genetically encoded Ca^2+^ reporter, GCaMP (Barnstedt et al., 2016). Furthermore *D. melanogaster* research has demonstrated that both the Kenyon cells, which constitute the insect memory center called the mushroom body, and their output neurons which mediate memory valence, are nicotinic (Aso et al., 2014; Barnstedt et al., 2016). These discoveries in *D. melanogaster* along with findings in pollinators, suggest that targeting nAChRs with insecticides in order to compromise their function will disrupt plasticity mechanisms causing cognitive deficits. It is expected that these will affect the non-target pollinator more, as it has a greater cognitive workload to perform its life cycle and pollinator services compared to a pest, such as an aphid which is just an essentially sedentary feeder on its food source.

## Circadian Rhythms

The circadian clock is integral to foraging efficiency in pollinators. Many aspects of floral resource availability, such as flower opening, scent release and nectar production are time-of-day-dependant (Bloch et al., 2017). This has led to foragers possessing an impressive ability to encode information and communication temporally (Beling, 1929). Honeybee foragers are able to learn to forage accurately at multiple feeders which are rewarding at different times of day (Van Nest et al., 2018), as little as 20 min apart (Koltermann, 1971). The time-of-day information is used along with other features such as color and odor to create a ‘*gestalt*,’ which is a robust memory of the rewarding resource. The time of reward is an integral part of this *gestalt*; if it is not reliable, learning success decreases by 9% (Bogdany, 1978). The circadian clock also directly affects their ability to learn; honeybees learn novel, rewarding odors better in the morning (Lehmann et al., 2011). This is thought to help them find new foraging patches, as most flowers are nectar-rich in the morning (Bloch et al., 2017). Likewise, *Drosophila* has been shown to demonstrate time-of-day memories and circadian change in olfaction and olfactory memory (Lyons and Roman, 2009; Chouhan et al., 2015; Flyer-Adams et al., 2020), showing that these behaviors, underlying mechanisms and potential impacts of neonicotinoids can be modeled using this system.

This circadian time keeping also allows honeybees to navigate *via* the sun compass and to communicate resource availability through the waggle dance (Beer et al., 2018). The waggle dance uses the sun’s azimuth as a landmark, requiring the dancer to know the position of the sun while dancing inside the darkness of the colony (Von Frisch and Chadwick, 1967). Some ‘marathon dancers’ continue to dance for hours, accurately shifting the angle of their dance to match that of the moving sun. Furthermore, honeybees also experience ‘jetlag’; if moved between time zones, foragers initially miscommunicate, using the azimuth from the last time zone rather than the current one (Renner, 1959). Clearly, bees have a time-in-place memory, an inner sense of time, and memory of the position and quality of the food sources necessary for foraging. This clearly demonstrates the interdependence of circadian rhythms and the huge cognitive feats pollinators must complete to perform their services and life cycle, all of which are negatively impacted by neonicotinoids.

Utilizing knowledge of the occurrence of nAChR receptor signaling in the clock in *D. melanogaster* (Wegener et al., 2004; Schlichting et al., 2019), and thus hypothesizing that the clock may be a target of neonicotinoids, a recent study explored the effect of neonicotinoids on circadian rhythmicity in *D. melanogaster*. Field-relevant concentrations of the banned neonicotinoids: imidacloprid, clothianidin and thiamethoxam all disrupted the circadian clock, reducing behavioral rhythmicity and increasing the proportion of activity that occurred during the night, while the non-banned thiacloprid left circadian behavior largely intact (Tasman et al., 2021). RNAi-mediated knockdown of either of the neonicotinoid susceptible nAChR subunits Dα1 and Dβ2 in the clock cells of the brain was sufficient to produce changes to rhythmicity mirroring those seen in neonicotinoid-induced flies, suggesting a role for these subunits in the clock and its disruption *via* neonicotinoids. Exposure of clock Dα1 and Dβ2 knockdown flies to imidacloprid or clothianidin (50 μg/L) caused no further change to rhythmicity, confirming the role of these subunits in the clock in mediating the effects of the neonicotinoids on circadian rhythms (Tasman et al., 2021). Both exposure to imidacloprid or clothianidin (50 μg/L) or knockdown of Dα1 or Dβ2 also prevented circadian plasticity and cycling of the availability of the circadian neuropeptide pigment dispersing factor PDF in the dorsal projections of the clock neuron small Lateral Neuron ventral (sLNv) (Tasman et al., 2021). The sLNv’s are a vital component of the clock, in which rhythmic expression of clock genes persists in constant conditions. Therefore these neurons are considered the pacemaker neurons, whose molecular clock regulates rhythmic behavior via rhythmic release of PDF and where day/night changes in sLNv complexity communicate time of day information to downstream clocks (Dubowy and Seghal, 2017). Therefore this study demonstrates that neonicotinoid exposure disrupts the day/night differences in clock neuron synaptic PDF neuropeptide availability and structural plasticity and decreases circadian rhythms. Interestingly, recent work suggests circadian changes in PDF signaling from the sLNv to the mushroom body may mediate time-of-day effects on *Drosophila* olfactory shock memory (Flyer-Adams et al., 2020). Therefore the disruption of sLNv synaptic PDF neuropeptide levels and structural plasticity may contribute to or be responsible for the deficits in mushroom body mediated olfactory shock memory seen. It would be interesting to test this in flies and see if the same circuitry mediates time of day information to the bee mushroom body memory circuit, explaining time-of-day effects on memory and the waggle dance as well as the effects of neonicotinoids in each.

Following on from this work, closely aligned lab experiments using *B. terrestris* showed that the same sub-lethal concentration of imidacloprid (10 μg/L) caused an analogous reduction in locomotor rhythmicity and foraging rhythmicity, increasing the proportion of mistimed foraging during the night (Tasman et al., 2020). Furthermore, similar effects were seen in honeybee, with 70 μg/L of clothianidin or thiamethoxam causing an increase in the proportion of arrhythmic honeybee foragers in normal light:dark and constant darkness (Tackenberg et al., 2020). These effects were made worse by putting the honeybees in constant light, suggesting that the effect of neonicotinoids on pollinators will be made worse by other forms of manmade activity, such as constant light pollution and climate change (Gilburn et al., 2015; Wood and Goulson, 2017; Fournier-Level et al., 2019; Goulson, 2019; Soroye et al., 2020; Wagner, 2020). The authors went on to show that *Drosophila* expressing GCaMP in the PDF expressing large Lateral Neuron ventral (l-LNv) clock neurons showed a transient (∼1 min) increase in Ca^2+^ dependent fluorescence with clothianidin (4 μg/L) (Tackenberg et al., 2020). This suggests that very low field-relevant concentrations of neonicotinoids can cause an increase in clock neuronal activity or intracellular Ca^2+^ (Tackenberg et al., 2020), which then causes an increase in synaptic PDF abundance and terminal complexity across the day, driving the loss of circadian rhythms and day/night sleep which resulted from as little as 1 μg/L clothianidin (Tasman et al., 2021). These studies suggest that sub-lethal effects of neonicotinoids are mediated by nAChRs containing Dα1 and Dβ2 subunits and that sleep and circadian rhythms may be the most sensitive behaviors to neonicotinoids and may also contribute to memory deficits. These studies clearly illustrate how knowledge of the occurrence and behavioral role of nAChRs in *D. melanogaster* can be utilized to identify novel sub-lethal effects of neonicotinoids and their mechanisms of actions which can then inform parallel studies in pollinators.

## Sleep

Sleep is controlled by two drives, the circadian clock, which dictates the timing of sleep and waking, and the sleep homeostat, which signals sleep need (Tononi and Cirelli, 2003; Fisher et al., 2013; Allada et al., 2017). Sleep is a universal, evolutionarily conserved and vital process, with extreme sleep deprivation being lethal. It is important for metabolism, endocrine, immunity, muscular-skeletal and brain function, for instance homeostatically regulating synaptic plasticity, aiding in behaviors such as memory (Donlea et al., 2011; Beyaert et al., 2012; Zwaka et al., 2015). Deep sleep is important for memory consolidation, with honeybees who are exposed to a trial odor during deep sleep performing better in olfactory learning tasks the next day (Zwaka et al., 2015), and sleep-deprived honeybees struggling to remember a novel navigational route from the day before (Beyaert et al., 2012). The timing of sleep is also important for tasks such as foraging, which needs to occur during the day, and brood care, which must occur round the clock (Nagari et al., 2019).

In *B. terrestris*, exposure to imidacloprid (10 μg/L) caused an increase in daytime and night-time sleep, with more daytime sleep episodes (Tasman et al., 2020). In *D. melanogaster*, exposure to imidacloprid, clothianidin, thiamethoxam or thiacloprid reduced and fragmented sleep, causing sleep to be composed of shorter and more frequent episodes (Tasman et al., 2021). RNAi-mediated knockdown of Dα1 and Dβ2 in the clock neurons of the brain reiterated the effects of neonicotinoids on sleep, suggesting a role for these subunits in sleep behavior and in mediating the effects of neonicotinoids on sleep. Another study also found Dα1 to be involved in sleep, with loss of this subunit causing loss of sleep, shorter sleep episodes in the night, and an increase in both the number of episodes and the amount of total sleep in the day (Somers et al., 2017). The subunit Dα4 also appears to mediate sleep, with a loss of function mutation in this subunit causing a reduction and fragmentation of sleep (Shi et al., 2014). Finally, clothianidin and thiamethoxam were also found to disrupt honeybee sleep, however they were considerably less sensitive than flies or bumblebees, requiring 140 μg/L of the drug to have an effect, with the neonicotinoids reducing total sleep and number of sleep episodes (Tackenberg et al., 2020). Further work will be required to determine the basis and significance of these species-specific sleep responses to neonicotinoids.

## Neuronal and Metabolic Functions

Therefore, as discussed the effects on neonicotinoids on neurons are broad, including changes in gene expression, mitochondrial function, neuronal excitability, mushroom body development, clock neuron synaptic neuropeptide abundance, plasticity and excitability (Badiou-Beneteau et al., 2012; Palmer et al., 2013; Moffat et al., 2015; Peng and Yang, 2016; Benzidane et al., 2017; Colgan et al., 2019; Tasman et al., 2020, 2021). Further mechanistic research using *Drosophila* has shown that 2.5 μg/L imidacloprid blocked nAChR agonist (e.g., carbachol) Ca^2+^ responses in larval neurons. Two hour exposure to 2.5 μg/L imidacloprid caused a significant increase in gut and brain levels of superoxide reactive oxygen species and decreased mitochondrial function and ATP levels (Martelli et al., 2020). This resulted in disruption of the lipid environment of metabolic tissues including brain and fat body (the insect equivalent of the liver) transcriptomic and lipidomic changes in expression inducing expression of genes related to the stress response, immunity and neurodegeneration. 20 days of exposure to field-relevant concentration of 4 μg/L imidacloprid resulted in loss of visual acuity, using electron microscopy this shown to cause retinal degeneration and vacuolization of glia. The neonicotinoid flies took longer to recover from shock and found it harder to mount an escape response. Interesting the later behavior was improved by co-treating the flies with an anti-oxidant. Loss of vision could impact many of the behaviors which neonicotinoids have been shown to affect, such as navigation (Beyaert et al., 2012), showing the importance of mechanistic studies possible in *Drosophila* to determine how neonicotinoids bring about their effects at the neuronal, metabolic and molecular level.

## Discussion

Neonicotinoid pesticides and analogous pesticides with the same mechanism of action represent an ongoing threat to beneficial insects such as pollinators (Woodcock et al., 2016). Due to the neurotoxic nature of these insecticides, they can cause a host of sub-lethal effects that can have a long-term detrimental impact on the survival and reproduction of pollinators such as bees. Despite the now well documented lethal and sub-lethal effects that neonicotinoids have on beneficial insects, they still account for 24% of the global insecticide market (Casida, 2018; Matsuda et al., 2020). They are implicated in the current dramatic losses in insects such as the 75% decrease in flying insect populations in the last 27 years (Goulson, 2019). This has potential catastrophic consequences. First, many of our best loved United Kingdom insect species (e.g., bees and butterflies), are greatly diminished in number, endangered or becoming extinct with knock-on effects for the ecosystem, aquatic life, birds and mammals (Wood and Goulson, 2017; Eng et al., 2019; Yamamuro et al., 2019). In addition, neonicotinoids have now been reported to be hazardous to humans (Han et al., 2018). Furthermore there are economic implications, with over a third of global crops (84% in Europe) dependent on pollinators (e.g., bees, butterflies, and flies) and whose services in the United Kingdom alone are valued at £430 million per year. The widely documented sub-lethal effects discussed in this review are not currently being picked up by the explicitly scored EU-mandated safety tests. Therefore there is a great need to rapidly identify and characterize the lethal and sublethal effects of current and especially novel pesticides before they are used in agriculture and released into the environment. We outlined the use of *D. melanogaster* and its versatile genetic and experimental tool-box for the rapid identification of these lethal and sub-lethal effects. Furthermore this model organism provides an expedited means of building a mechanistic understanding of these pesticides. This approach will facilitate the essential production of a knowledge base to aid in the creation of more pest or species-specific insecticides in the future.

As discussed, *D. melanogaster*, whose genome is particularly genetically tractable and well annotated, provides a valuable guide for the identification of genes and subsequent mutant and molecular characterization which easily translates to pollinators like honeybees and bumblebees, due to their close evolutionary relatedness. The molecular and functional homology of neonicotinoid signaling and metabolic genes seem particularly highly conserved between insects, and specifically the nAChRs targeted by neonicotinoids. By identifying sub-lethal neonicotinoid effects common between *D. melanogaster* and pollinators, such as learning, circadian rhythms, sleep, locomotion, and immune responses the genetic tools and established experimental assays and reagents available in *D. melanogaster* can then be used to rapidly characterize these behavioral and physiological responses. Many of the genetic findings and tools developed in *Drosophila* can then be translated to bees and butterflies, including the use of RNAi and CRISPR/Cas9 genome editing, as well as using *Drosophila* gene sequences to design primers, *in situ* probes and antibodies that work on other these insects.

Despite the advantages of using *D. melanogaster* as a genetic model, it should be used to enhance (rather than replace) experimentation in bees. As has been identified in some of the research reviewed here, a model can never fully replicate that which it models, and some behavioral effects of neonicotinoids, such as on sleep and gut microbiota, will differ between the two species. Exploring these differences can provide insight into the potential for species or pest-specific insecticides. Thus *D. melanogaster* will be most valuable when used in tandem with behavioral studies in bees and other pollinators providing rapid in-depth exploration and validation that is still not feasible in non-model organisms.

Given the ongoing tension between the need for food security balanced against the decline in pollinator populations we advocate the use of every possible tool route for identifying the potential dangers and attempting to find safer alternatives and propose *D. melanogaster* as a valuable tool for this ongoing work.

## Author Contributions

KT wrote the first draft of the review. SR and JH edited the review. JH secured the funding. All the authors contributed to the article and approved the submitted version.

## Conflict of Interest

The authors declare that the research was conducted in the absence of any commercial or financial relationships that could be construed as a potential conflict of interest.
